# Aquatic Exposure to Abscisic Acid Transstadially Enhances *Anopheles stephensi* Resistance to Malaria Parasite Infection

**DOI:** 10.3390/genes11121393

**Published:** 2020-11-24

**Authors:** Dean M. Taylor, Reagan S. Haney, Shirley Luckhart

**Affiliations:** 1 Department of Entomology, Plant Pathology and Nematology, University of Idaho, Moscow, ID 83844, USA; tayl3660@vandals.uidaho.edu (D.M.T.); hane2410@vandals.uidaho.edu (R.S.H.); 2 Department of Biological Sciences, University of Idaho, Moscow, ID 83844, USA

**Keywords:** abscisic acid, mosquito, malaria, *Anopheles*, *Plasmodium*, resistance, immunity, insulin-like peptide, nitric oxide

## Abstract

The ancient stress signaling molecule abscisic acid (ABA) is ubiquitous in animals and plants but is perhaps most well-known from its early discovery as a plant hormone. ABA can be released into water by plants and is found in nectar, but is also present in mammalian blood, three key contexts for mosquito biology. We previously established that addition of ABA to *Anopheles stephensi* larval rearing water altered immature development and life history traits of females derived from treated larvae, while addition of ABA to an infected bloodmeal increased resistance of adult female *A. stephensi* to human malaria parasite infection. Here we sought to determine whether larval treatment with ABA could similarly impact resistance to parasite infection in females derived from treated larvae and, if so, whether resistance could be extended to another parasite species. We examined nutrient levels and gene expression to demonstrate that ABA can transstadially alter resistance to a rodent malaria parasite with hallmarks of previously observed mechanisms of resistance following provision of ABA in blood to *A. stephensi*.

## 1. Introduction

Malaria is a vector-borne disease caused by protozoan parasites of the genus *Plasmodium* that are transmitted by *Anopheles* spp. mosquitoes. Based on disease-associated mortality, *Plasmodium falciparum* is the most important human parasite and one of five known malaria parasite species that threaten more than half the world’s population [1]. In 2016, the Indian malaria mosquito *Anopheles stephensi* was first recorded in Ethiopia [2], after earlier discovery in Djibouti [3], and has since become established in the region. The invasiveness of *A. stephensi* presents new challenges to the control of malaria as this anopheline is unique in its ability to breed in both natural bodies of water and in man-made containers found in urban environments [4]. Accordingly, the establishment of an urban malaria vector in East Africa is a serious and urgent health threat to the region and the continent [5].

Environmental factors have significant effects on mosquito development, adult fitness and vector competence. For example, the microbiota in adult mosquito midguts is influenced by bacteria present in the larval environment [6,7,8,9]. Notably, the midgut microbiota of adult *Aedes* and *Anopheles* spp. can modify vector competence to multiple pathogens [10,11,12]. The presence of animal detritus in the larval environment as compared to plant detritus, has been associated with reduced development time, increased adult survival and increased adult body mass of *Aedes albopictus* [13] and of three species of the *Anopheles gambiae* complex [14]. Competition and threat of predation in the larval environment can significantly impact larval development time, adult size, fecundity and adult behavior of species in multiple genera [15,16]. While many plant extracts have been tested and validated for bioactivity against mosquito larvae (reviewed in [17]), few studies have examined the effects of naturally present plant compounds, including plant hormones, in aquatic habitats on mosquito life history and biology, including adult resistance to pathogen infection. 

The isoprenoid abscisic acid (ABA), first discovered as a plant hormone in the 1960s and reviewed in [18], is released into water from inundated plants and from plant material [19,20], suggesting that mosquito oviposition sites could be associated with exposure of immature mosquito stages to ABA. To determine the potential significance of this exposure, we showed that the addition of ABA to rearing water at concentrations as low as 1 μM significantly accelerated *A. stephensi* larval development, increased pupal mortality, decreased lifespan and decreased fecundity through three gonotrophic cycles in adult female mosquitoes derived from treated larvae relative to controls [20]. 

In addition to aquatic exposure, mosquitoes can be exposed to ABA as adults through the ingestion of blood and nectar, both of which are sources of this ancient stress signaling molecule [21,22,23]. Accordingly, we tested oral delivery of ABA in a *P. falciparum*-infected bloodmeal to *A. stephensi*. The ABA-supplemented bloodmeal significantly reduced infection prevalence and mean numbers of parasites per infected mosquito [24]. Treatment was also associated with transient decreases (<24 h) in the expression of *insulin-like peptide* (*ilp*) *3* and *ilp4* and concurrent increases in midgut *nitric oxide synthase* (*nos*) expression and anti-parasite NO [25]. Given that *P. falciparum* infection of *A. stephensi* induces the expression of *ilp3* and *ilp4*, which suppresses the host immune response [26], we reasoned that ABA mediates its effects on parasite infection in part via activation of insulin/insulin-like growth factor signaling (IIS). Interestingly, bloodmeal-delivered ABA reduced *P. falciparum* development in *A. stephensi* without significant changes in the adult female lifespan or fecundity in the first gonotrophic cycle [24,25]. In contrast to the transient effects of ABA treatment of adult *A. stephensi*, ABA treatment of *A. stephensi* larvae resulted in effects that were durable and transstadial [20]. 

Notable among the effects of ABA in adult female, *A. stephensi* derived from treated larvae were reductions in *Vitellogenin* (*Vg*) transcript expression of 90% (100 µM ABA) and 98.5% (1 µM ABA) compared to controls at 24 h following a blood meal [20]. Under normal conditions, the yolk protein precursors Vg and lipophorin (Lp) are induced after blood feeding. Lp is comprised of Apolipophorin (ApoLp)-I/II and is required for *Vg* expression [27] and for transport of lipids to various tissues and developing oocytes. A third gene product, ApoLp-III, has been identified in both the African malaria mosquito *Anopheles gambiae* and in *A. stephensi*, although the involvement of this protein in lipid transport has not been definitively demonstrated [27,28,29,30]. Vg and Lp, in addition to their roles in reproduction, together with ApoLp-III have been associated with effects on *Plasmodium* spp. ookinete establishment and in transporting nutrients to developing parasite oocysts [27,31]. Mendes et al. [32] showed that RNAi-induced silencing of *Lp* reduced *P. falciparum* oocyst density in *A. gambiae*. Subsequent studies showed that the depletion of Lp and the associated limitation of lipid resources for parasite development decreased the size of *P. falciparum* oocysts and the density of salivary gland sporozoites [31]. The absence of Lp and Vg has also been shown to increase binding of the anti-parasite thioester-containing protein 1 (TEP1) to *Plasmodium berghei* ookinetes in *A. gambiae* [27]. While silencing of *apoLp-III* had no effect on *P. berghei* or *P. falciparum* development in *A. gambiae*, RNAi-induced gene silencing of *apoLp-III* in *A. stephensi* led to increased *nos* expression at 24 h post blood meal and reduced numbers of *P. berghei* oocysts [30,32]. 

Here, we sought to examine the possibility that ABA treatment of *A. stephensi* larvae could alter malaria parasite infection in adult females derived from treated larvae based on the above findings and some key prior observations. First, we previously reported that ABA treatment of *A. stephensi* larvae resulted in a large and significant decrease in *Vg* expression in adult female mosquitoes [20], suggesting that adult *A. stephensi* derived from treated larvae may be more resistant to parasite infection than females derived from untreated, control larvae. Further, given that Lp is required for *Vg* expression [27], resistance that is induced by repressed *Vg* expression might be associated with decreased Lp levels and, therefore, decreased lipid transport and catabolism [20,27]. Second, in light of the comprehensive and durable effects of ABA larval treatment, we hypothesized that adults from ABA-treated larvae might exhibit changes in *ilp* expression that were similar to changes in *ilp* expression observed in female *A. stephensi* provisioned with ABA in a bloodmeal [25]. This last question was of particular importance given our observations that ilps and IIS regulate *P. falciparum* development in *A. stephensi* [26,33,34,35,36,37,38], that ilps and IIS regulate *nos* expression [26,38,39] and that *nos* expression and anti-parasite NO, in particular, were induced to high levels in adult *A. stephensi* within 4–6 h after ingestion of an ABA-supplemented bloodmeal to control *P. falciparum* infection [24,25]. Third, TEP1-dependent parasite killing is effective against *P. falciparum* and *P. berghei* in *A. gambiae* [40] and is mediated by a complex of the leucine-rich repeat proteins LRIM1 and APL1C that facilitate TEP1 recognition of parasites and subsequent killing by either lysis or melanization [41]. While this pathway has been studied predominantly in *A. gambiae*, published observations on the lack of an effect of silencing *A. stephensi* LRIM1 on *Plasmodium yoelii yoelii* 17XNL infection [42] indicate that this pathway is not operative in the regulation of this mosquito-parasite combination. 

Accordingly, to compare and contrast with our previous studies of the effects of bloodmeal-delivered ABA on *P. falciparum* infection in *A. stephensi* [24,25], we examined *P. y. yoelli* 17XNL infection in female *A. stephensi* derived from ABA-treated and control larvae. Relative to females derived from control larvae, female *A. stephensi* derived from ABA-treated larvae were significantly more resistant to parasite infection. Patterns of lipid content and *apoLp-III* expression relative to controls were not significantly altered in adult females derived from ABA-treated larvae and, therefore, were not associated with infection resistance. Significant ABA-dependent changes in *ilp* and *nos* expression, however, were associated with resistance, highlighting signaling and effector biology that underlie both transstadial and intrastadial effects of ABA on *A. stephensi* resistance to distinct species of *Plasmodium*. 

## 2. Materials and Methods 

### 2.1. Mosquito Colony Rearing and Maintenance 

*Anopheles stephensi* Liston (Indian wild-type strain) were reared and maintained in temperature controlled chambers at 27 °C and 80% humidity with a 16/8-h light/dark cycle. Adult mosquitoes were housed in 1 ft^3^ wire mesh cages and provided continuous access to 10% sucrose-soaked cotton pads. At three days post-eclosion, adult female mosquitoes were allowed to feed on live mice sedated with ketamine (50 mg/kg) and xylazine (5 mg/kg) in sterile saline. Use of animals, including parasite infections described in Section 2.2. below, was in strict accordance with the recommendations in the Guide for Care and Use of Laboratory Animals of the National Institutes of Health and the Institutional Animal Care and Use Committees at the University of Idaho under protocol IACUC-2020-10 (approved on 30 March 2020). Mosquitoes were provided shallow cups of water to oviposit 48 h after blood feeding. Eggs were gently washed into 5 L Nalgene pans with shallow water. Larvae were maintained in 5 L Nalgene pans on a solution of 2% powdered fish food (Sera Micron, Heinsberg, Germany) and baker’s yeast in a 2:1 ratio for the first 3 days followed by Game Fish Chow pellet food (Purina, Arden Mills, MN, USA) until pupation. Adult mosquitoes were collected for experiments within 12 h post-eclosion and housed in screened cartons. 

### 2.2. Mouse Infection and Parasite Transmission to A. stephensi

Outbred CD1 mice (6–8 week old, Jackson Laboratories, Bar Harbor, ME, USA) were infected with 5 × 10^6^ *P. y. yoelii* 17XNL-infected red blood cells (RBCs). Parasitemia was measured daily following infection and mice were monitored for signs of disease. Thin film blood smears stained with 1% Giemsa were used to record parasitemia as the percentage of infected RBCs divided by the total number of RBCs. At three days post-infection mice were checked for microgamete exflagellation. When exflagellation was observed at more than 10 events per 40x field, mice were anesthetized with ketamine (50 mg/kg) and xylazine (5 mg/kg) and exposed to the bites of 3–5 day old female *A. stephensi* for 15 min then promptly removed. Non-fed females were removed and discarded. Infected mosquitoes were maintained on 10% sucrose pads at 24 °C and 80% humidity. At 10 days post-feeding mosquito midguts were dissected and prepared for analysis only from females with fully developed eggs. Midguts were stained in 1% mercurochrome for 3 min to count oocysts for infection prevalence (the presence of at least one midgut oocyst) and intensity (mean number of oocysts per infected midgut). Transmission studies were completed with two separate biological cohorts of adult female *A. stephensi* derived from ABA-treated and control larvae (Section 2.3) and separate sets of infected mice.

### 2.3. Treatment of Mosquito Larvae with ABA

Larvae were collected at 36 h following transfer of eggs into Nalgene pans and separated into four treatment groups. For each treatment group, 100 larvae were placed in 500 mL polypropylene Nalgene containers with 200 mL water with or without 1, 10 or 100 μM ABA (Caisson Labs, Smithfield, UT, USA) as described [20]. Due to the light sensitivity of ABA, 50 mL of water from each container was removed daily as described and replaced with freshly made ABA-supplemented water to yield a final concentration of 1, 10 or 100 μM ABA [20]. These concentrations were selected based on levels of ABA previously detected in water from inundated plant material under lab conditions. Specifically, levels of ABA detected ranged from 0.5 μM to nearly 3 μM through 48 h after plant material was added to water [20]. Larvae were fed as described in Section 2.1. and maintained through pupation and eclosion to adults. For infection studies (Section 2.2), qRT-PCR assays (Section 2.4) and triacylglycerol quantification (Section 2.5), ABA-treated and control larvae were prepared from 2–5 separate biological cohorts of *A. stephensi*. 

### 2.4. qRT-PCR Assays

Relative transcript levels were determined for *A. stephensi nos*, *ilps 1–5* and *apoLp-III* using previously published primers [20,25,30]. All data were normalized to transcript levels of *A. stephensi ribosomal protein s7* (*rps7*) [20]. Adult female mosquitoes (as indicated in figure legends) were immobilized by briefly freezing at −20 °C and pools of five adults were placed in 500 μL Trizol (Invitrogen, Carlsbad, CA, USA) for RNA extraction according to manufacturer’s instructions. cDNA was synthesized using QuantiTect reverse transcriptase kit (Qiagen, Hilden, Germany) according to manufacturer’s instructions. cDNA concentrations were adjusted to 250 ng/μL with molecular grade water. Target gene data were normalized to *rps7* data and reported as relative fold change. Biological replicates of these transcript expression analyses were completed with three separate cohorts of adult female *A. stephensi* derived from ABA-treated and control larvae (Section 2.3). Individual amplification reactions were performed in triplicate to confirm amplification consistency. 

### 2.5. Triacylglycerol (TAG) Quantification

The assay for TAG quantification was adapted from Tenneson et al. [43]. For these studies, two adult female *A. stephensi* were collected 1, 2, 3 and 4 days following eclosion from each larval treatment group and placed on ice. Females were rinsed several times in cold 1x phosphate-buffered saline (PBS) to remove external debris and then were transferred to a 1.5 ml centrifuge tube. A total of 100 μL of PBS + 0.05% Tween 20 (PBST) was added to each sample, followed by rapid homogenization on ice using a Dounce tissue grinder and heating for 10 min at 70 °C. Heat-inactivated samples were frozen at −80 °C until assayed. Aliquots of 20 μL of each thawed sample were placed in two tubes, one with 20 μL PBST to measure free glycerol and one with 20 μL triglyceride reagent T2449 (Sigma-Aldrich, St. Louis, MO, USA) to measure glycerol from digested triglyceride. Tubes were incubated at 37 °C for 60 min, then added to a 96-well plate. A total of 100 μL of free glycerol reagent F6428 (Sigma-Aldrich, St. Louis, MO, USA) was added to each well and incubated at 37 °C for 5 min. Absorbance at 540 nm was measured on a SpectraMax Paradigm plate reader (Molecular Devices, San Jose, CA, USA). TAG concentrations were calculated by subtracting free glycerol concentrations from total glycerol concentrations in digested samples. TAG concentrations were back calculated using a linear glycerol standard curve (2.5 mg/mL triolein equivalent glycerol standard G7793; Sigma-Aldrich, St. Louis, MO, USA) from 0.25 to 1.0 mg/mL. Replicates of TAG quantification studies were completed with 4–5 separate biological cohorts of *A. stephensi* derived from ABA-treated and control larvae (Section 2.3).

### 2.6. Statistical Analyses

All statistical analyses were performed using R statistical software version 3.5.3 and all figures were created using ggplot2 package within R. Infection prevalence data were analyzed using the G-Test likelihood ratio test of independence. Infection intensity data were analyzed by ANOVA and *post hoc* Tukey’s test. Lipid levels were normalized to control levels and analyzed by ANOVA. Data from qRT-PCR assays were normalized by 2^–ΔΔCt^ (log_2_ transformed for negative fold change) and analyzed by ANOVA. Differences were considered significant at α ≤ 0.05.

## 3. Results

### 3.1. Relative to Controls, Female A. stephensi Derived from ABA-Treated Larvae Were Significantly More Resistant to P. y. yoelii 17XNL Infection 

In previous work, we showed that ABA treatment of *A. stephensi* larvae resulted in comprehensive and durable changes to adult *A. stephensi* biology, including significant suppression of *Vg* expression at 24 h following blood feeding [20]. Based on these observations and the effects of Vg, Lp and ApoLp-III on malaria parasite development and mosquito immunity noted above, we sought to examine the success of *P. y. yoelii* 17XNL development in *A. stephensi* derived from ABA-treated and control larvae. 

**Figure 1:**
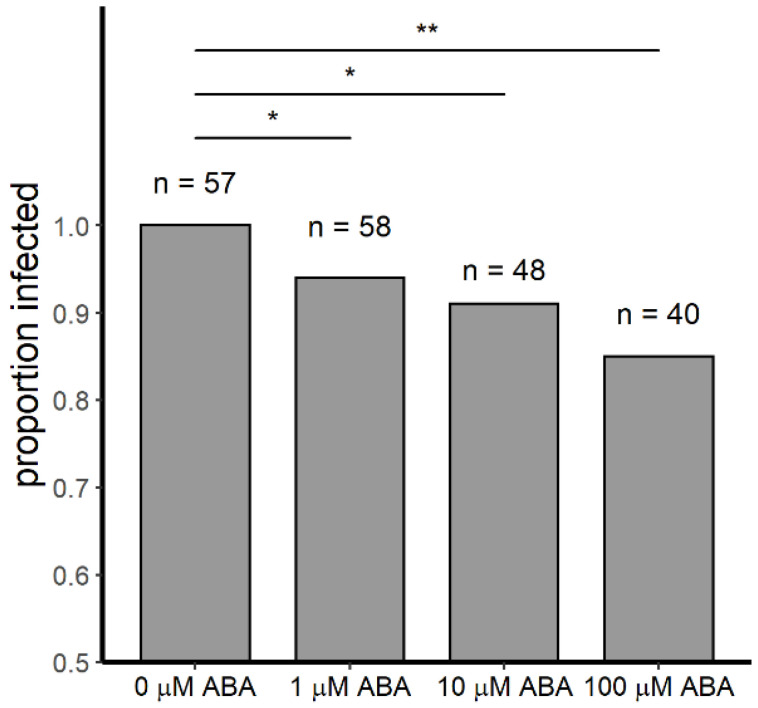
Proportions of female *A. stephensi* infected with at least one *P. y. yoelii* 17XNL oocyst were reduced in mosquitoes derived from ABA-treated larvae relative to controls. For these studies, 3–5 day old female mosquitoes were used for infection. Compared to controls (0 µM ABA), infection prevalence in female mosquitoes derived from larvae treated with 1 µM ABA was reduced by 5.2% (*n* = 58, *p* = 0.041), in females derived from larvae treated with 10 µM ABA it was reduced by 8.3% (*n* = 50, I = 0.011), and in females derived from larvae treated with 100 µM ABA it was reduced by 15% (*n* = 40, *p* = 0.001). Data represent two biological replicates with separate cohorts of *A. stephensi*. Data were analyzed by the G-test likelihood ratio test of independence. * *p* ≤ 0.05, ** *p* ≤ 0.001.

**Figure 2:**
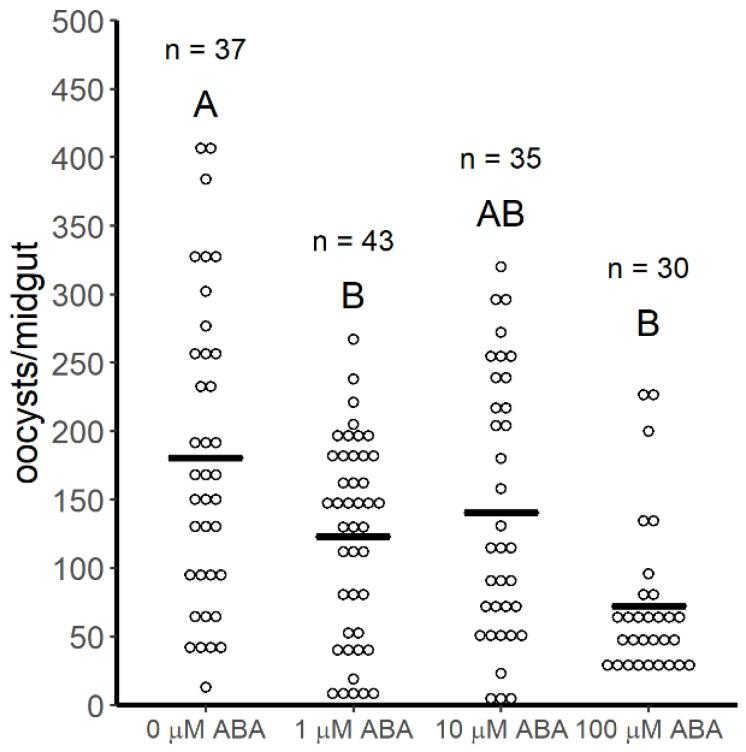
Infection intensity (mean *P. y. yoelii* 17XNL oocysts per infected midgut from mosquitoes in Figure 1) was reduced in *A. stephensi* derived from ABA-treated larvae relative to controls. For these studies, 3–5 day old female mosquitoes were used for infection. Mean numbers of oocysts per infected midgut (black bars) were significantly reduced (ANOVA *p* = 2.204 × 10^−5^) relative to control mosquitoes (0 µM ABA, 181 oocysts per midgut) in females derived from larvae treated with 1 μM ABA (123 oocysts per midgut, Tukey *p* = 0.020) and in females derived from larvae treated with 100 μM ABA (83 oocysts per midgut, Tukey *p* < 0.001). While there was a trend toward reduced infection intensity in females derived from larvae treated with 10 μM ABA, this difference was not significant relative to controls. Data represent two biological replicates with separate cohorts of *A. stephensi*. Data were analyzed by ANOVA and Tukey’s post hoc test. Different capital letters indicate differences among control and treatment groups.

In our studies, infection prevalence in 3–5 day old control female *A. stephensi* was 100% in both replicates (*n* = 57). Further, infection intensities (mean oocysts per midgut) in control females were not significantly different between replicates (Student’s *t*-test, *p* = 0.42), so replicate data were combined for analysis. Compared to controls, infection prevalence in female mosquitoes derived from larvae treated with 1 µM ABA was reduced by 5.2% (*n* = 58, *p* = 0.041), in females derived from larvae treated with 10 µM ABA it was reduced by 8.3% (*n* = 50, *p* = 0.011), and in females derived from larvae treated with 100 µM ABA it was reduced by 15% (*n* = 40, *p* = 0.001) (Figure 1). Moreover, mean numbers of oocysts per infected midgut were significantly reduced (ANOVA *p* = 2.204 × 10^−5^) relative to control mosquitoes (181 oocysts per midgut) in females derived from larvae treated with 1 μM ABA (123 oocysts per midgut, Tukey *p* = 0.020) and in females derived from larvae treated with 100 μM ABA (83 oocysts per midgut, Tukey *p* < 0.001) (Figure 2). While there was a trend toward reduced infection intensity in females derived from larvae treated with 10 μM ABA, this difference was not significant relative to control mosquitoes (Figure 2).

### 3.2. TAG Concentration and Expression of apoLp-III Were Unaltered in Adult Female A. stephensi Derived from ABA-Treated Larvae Relative to Mosquitoes Derived from Control Larvae

**Figure 3:**
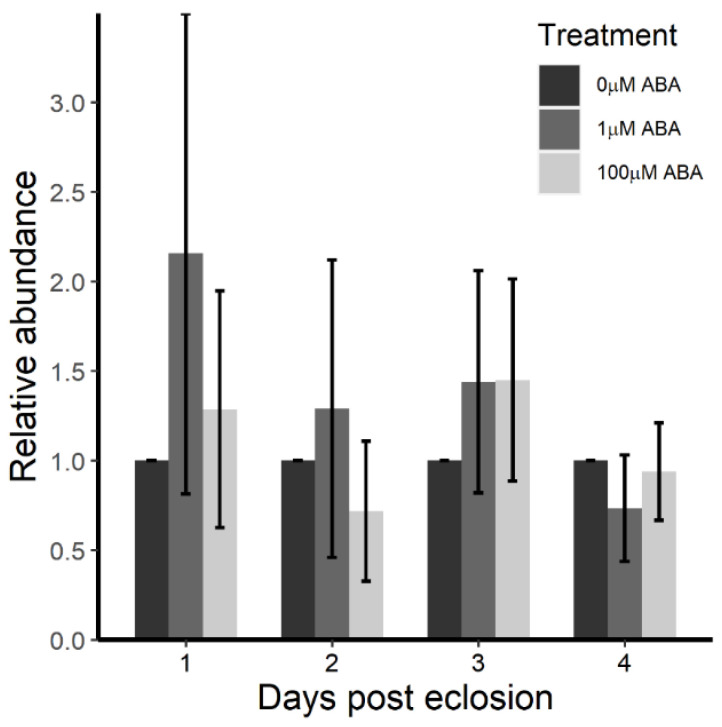
Content of the neutral lipid triacylglycerol (TAG) in sugar-fed, adult female *A. stephensi* derived from control and ABA-treated larvae did not differ through four days following eclosion. Relative to controls (0 µM ABA), there were no differences in TAG abundance in females derived from ABA-treated larvae at day 1 (*F* = 1.931, *p* = 0.199), day 2 (*F* = 2.420, *p* = 0.132), day 3 (*F* = 1.416, *p* = 0.269) or day 4 (*F* = 2.440, *p* = 0.139). Data were analyzed by ANOVA and are shown as mean ± SE across 4–5 biological replicates with separate cohorts of *A. stephensi*.

Given the repression in *Vg* transcript levels in female *A. stephensi* derived from ABA-treated larvae [20] and the dependency of induced *Vg* expression on elevated Lp [27], we reasoned that reduced Lp levels might be associated with reduced lipid transport to body tissues in adult females derived from ABA-treated larvae. In anophelines, reserves at adult emergence are much lower than for culicines and, therefore, anophelines are nearly entirely dependent on lipid biosynthesis from resources ingested after eclosion [44]. In the absence of blood-derived lipids, provisioned sugar or nectar is converted to triacylglycerol (TAG) as the primary storage lipid. In female *A. gambiae*, Lp transports TAG and a variety of neutral lipids to tissues where TAG is hydrolyzed to diacylglycerol (DAG) and, in some cases, to free fatty acids (FFAs), to be used as energy sources for flight, previtellogenic ovarian development, immunity and other resource-consuming processes [45]. In addition to these functions, *A. gambiae* Lp and its lipid cargo are taken up by developing *P. falciparum* oocysts [31], suggesting that reductions in *Vg* expression and, consequently, Lp and TAG levels, could explain reduced parasite infection success in female *A. stephensi* derived from ABA-treated larvae (Figure 1 and Figure 2). Based on these observations, a significant reduction in Lp after adult eclosion would be evident as a failure to transport TAG to various tissues for hydrolysis to DAG and FFAs. Accordingly, low Lp levels in adult female *A. stephensi* derived from ABA-treated larvae would be predicted to be associated with accumulated TAG compared to females derived from control larvae where TAG catabolism is unabated. In contrast to our expectations, however, we did not observe significant differences in TAG levels in the first four days following adult emergence in sugar-fed female *A. stephensi* derived from ABA-treated versus control larvae. Day 1 (*F* = 1.931, *p* = 0.199), day 2 (*F* = 2.420, *p* = 0.132), day 3 (*F* = 1.416, *p* = 0.269) and day 4 (*F* = 2.440, *p* = 0.139) (Figure 3).

**Figure 4:**
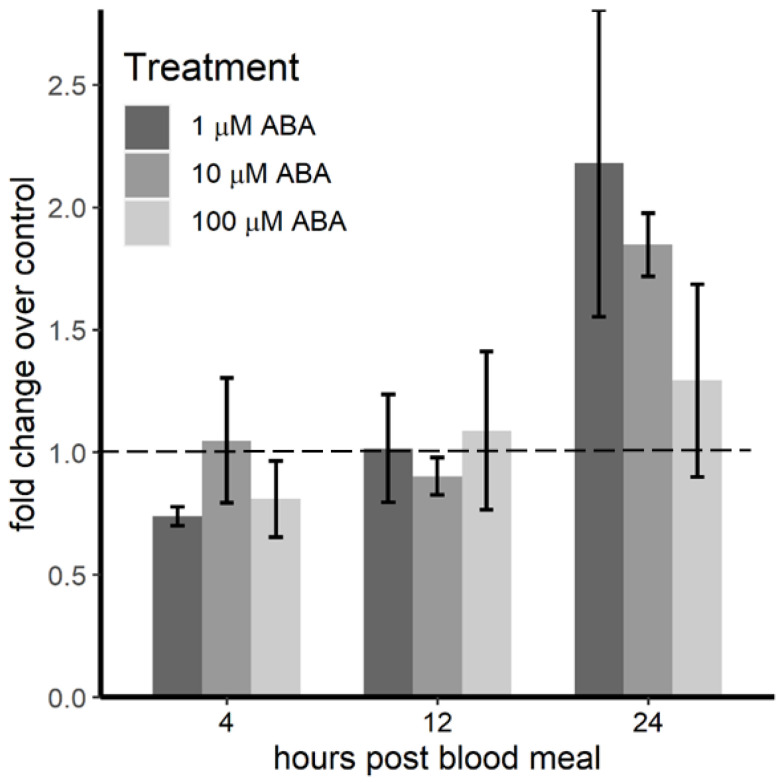
*ApoLp-III* transcript expression in *P. y. yoelii* 17XNL-infected *A. stephensi* derived from control and ABA-treated larvae did not differ through 24 h post-infection. For these studies, 3–5 day old female mosquitoes were used for infection and analysis of *apoLp-III* expression. Transcript levels were quantified by qRT-PCR using the 2^–ΔΔCt^ method; controls (0 µM ABA) were set to 1 (dashed line). Relative to controls, there were no significant differences in *apoLp-III* expression at 4 h (*F* = 0.641, *p* = 0.610), 12 h (*F* = 0.085, *p* = 0.966) or 24 h (*F* = 1.377, *p* = 0.318) post-infection in females derived from larvae treated with 1, 10 or 100 µM ABA. Data were analyzed by ANOVA and are shown as mean ± SE for three biological replicates with separate cohorts of *A. stephensi*.

In addition to ApoLp-I/II, ApoLp-III has been associated with lipid transport in insects, but its involvement in this process in *A. stephensi* has not been definitively demonstrated. However, *apoLp-III* expression is strongly induced by *P. berghei* infection in *A. stephensi*, consistent with ApoLp-III control of infection [30]. Accordingly, we examined expression of *apoLp-III* in *P. y. yoelii* 17XNL-infected *A. stephensi* with the expectation that *apoLp-III* expression might be induced in females derived from ABA-treated larvae relative to females derived from control larvae. However, our results showed no significant differences in *apoLp-III* transcript expression between females derived from control larvae and females derived from ABA-treated larvae at 4 h (*F* = 0.641, *p* = 0.610), 12 h (*F* = 0.085, *p* = 0.966) or 24 h (*F* = 1.377, *p* = 0.318) after a blood meal (Figure 4).

### 3.3. Insulin-Like Peptide (ilp) Gene Expression in Female A. stephensi Derived from ABA-Treated Larvae Was Reduced Relative to Females Derived from Control Larvae 

Given the comprehensive effects of ABA treatment of *A. stephensi* larvae on adult female lifespan, reproduction and immunity to infection [20], parameters that we and others have causally linked to ilps and IIS (reviewed in [46]), and the transient changes in *ilp3* and *ilp4* expression that followed provisioning of ABA in a bloodmeal to adult female *A. stephensi* [25], we sought to examine *ilp* expression in adult female *A. stephensi* after emergence from ABA-treated and control larvae. Specifically, the durability and transstadial effects of larval treatment with ABA suggested that *ilp* expression might be altered in adult female *A. stephensi* derived from ABA-treated larvae compared to controls. Moreover, significantly reduced *ilp* expression would be consistent with cause-and-effect increases in immune gene expression and anti-parasite NO synthesis that control parasite infection in *A. stephensi* [24,25,26]. 

**Figure 5:**
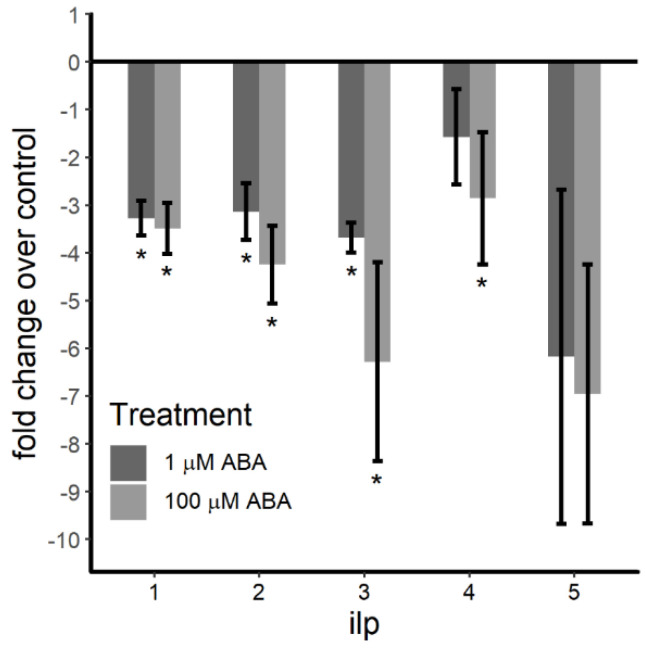
*Insulin-like peptide* (*ilp*) expression levels in 3-day-old female *A. stephensi* derived from ABA-treated larvae were reduced relative to females derived from control larvae. Transcript levels were quantified by qRT-PCR using the log_2_(2^–ΔΔCt^) method; untreated controls (0 µM ABA) were set to 0. Transcript levels were reduced for *ilp1* (*F* = 18.83, *p* = 0.003), *ilp2* (*F* = 9.02, *p* = 0.012), *ilp3* (*F* = 60.92, *p* < 0.001) and *ilp4* (*F* = 4.26, *p* = 0.05). Compared to controls, expression levels for *ilp1* (*p* = 0.003), *ilp2* (*p* = 0.035) and *ilp3* (*p* < 0.001) were significantly reduced in mosquitoes derived from 1 µM ABA-treated larvae. In females derived from 100 µM ABA-treated larvae, transcript levels were reduced relative to controls for *ilp1* (*p* = 0.006), *ilp2* (*p* = 0.012), *ilp3* (*p* < 0.001) and *ilp4* (*p* = 0.047). However, transcript levels for *ilp5* were not significantly reduced relative to control in either group of females derived from ABA-treated larvae (*F* = 0.259, *p* = 0.774). Data were analyzed by ANOVA and are shown as mean ± SE for three biological replicates with separate cohorts of *A. stephensi*. * *p* ≤ 0.05.

To test these hypotheses, we measured *ilp 1–5* transcript levels in female *A. stephensi* derived from ABA-treated and control larvae. Transcript levels were reduced for *ilp1* (*F* = 18.83, *p* = 0.003), *ilp2* (*F* = 9.02, *p* = 0.012), *ilp3* (*F* = 60.92, *p* < 0.001) and *ilp4* (*F* = 4.26, *p* = 0.05). Compared to controls, expression levels of *ilp1* (*p* = 0.003), *ilp2* (*p* = 0.036) and *ilp3* (*p* < 0.001) were significantly reduced in mosquitoes derived from larvae treated with 1 µM ABA (Figure 5). In adult females derived from larvae treated with 100 µM ABA, we observed significant reductions in expression levels of *ilp1* (*p* = 0.006), *ilp2* (*p* = 0.012), *ilp3* (*p* < 0.001) and *ilp4* (*p* = 0.047) compared to controls. Neither group of female *A. stephensi* derived from ABA-treated larvae showed significant changes in expression of *ilp5* (F = 0.259, *p* = 0.774), however, compared to controls (Figure 5). 

### 3.4. Nitric Oxide Synthase (nos) Gene Expression in Female A. stephensi Derived from ABA-Treated Larvae Was Increased Relative to Mosquitoes Derived from Control Larvae 

In prior work, we established that *A. stephensi* targets *Plasmodium* spp. within the first 24 h of development in the midgut with inducible, anti-parasite NO synthesis to regulate infection [39,47]. We have also associated knockdown of *ilp4* with early induction of the immune effector genes *nos*, *apl1*, *tep1* and *lrim1* (1–6 h after infection) and knockdown of *ilp3* with increased later expression of the same genes (24 h after infection) in *P. falciparum*-infected *A. stephensi* [26]. Among these gene products, APL1, LRIM1 and TEP1 are associated with TEP1-mediated parasite killing, a process that does not appear to be functional during infection of *A. stephensi* with *P. y. yoelii* 17XNL [42]. Accordingly, our observations pointed to inducible *nos* expression as the most likely explanation for the observed effects on *P. y. yoelii* 17XNL infection (Figure 1 and Figure 2). 

**Figure 6:**
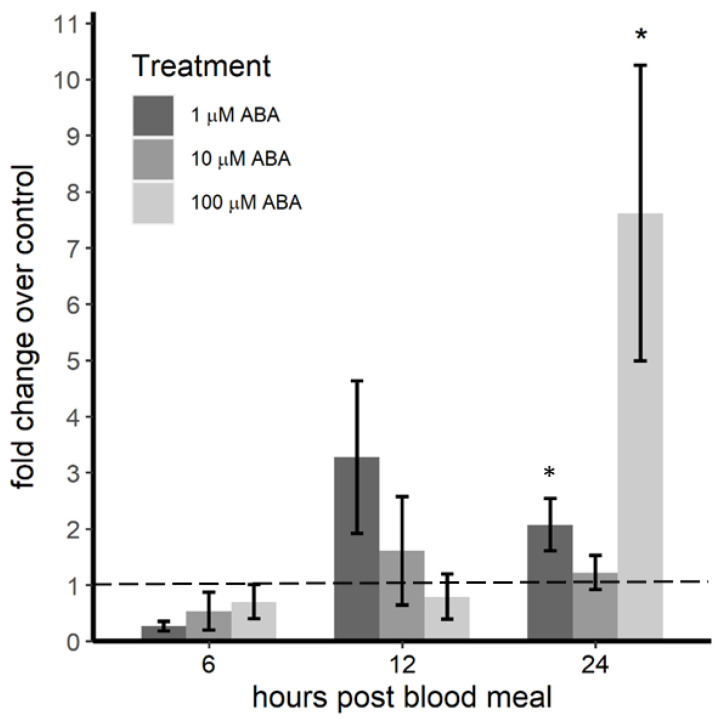
*Nos* expression in *P. y. yoelii* 17XNL-infected *A. stephensi* derived from ABA-treated larvae was induced at 24 h post-infection. Transcript levels were quantified by qRT-PCR using the 2^–ΔΔCt^ method; untreated controls (0 µM ABA) were set to 1 (dashed line). There were no significant changes in *nos* expression relative to controls at 6 h (*F* = 0.991, *p* = 0.483) or 12 h (*F* = 1.159, *p* = 0.390) post-infection in females derived from ABA-treated larvae. However, *nos* expression was increased compared to controls at 24 h (*F* = 4.795, *p* = 0.014) in *A. stephensi* derived from larvae treated with 1 μM ABA (Tukey *p* = 0.041) and 100 μM ABA (Tukey *p* = 0.016) but not 10 μM ABA (Tukey *p* > 0.05). Data are shown as mean ± SE across three biological replicates. Data were analyzed by ANOVA. * *p* ≤ 0.05.

To test this hypothesis, we examined *nos* expression in female *A. stephensi* derived from control larvae or larvae treated with 1, 10 or 100 µM ABA at 6, 12 and 24 h post-infection with *P. y. yoelii* 17XNL. We did not observe significant changes in *nos* expression relative to controls at 6 h (*F* = 0.991, *p* = 0.483) or 12 h (*F* = 1.159, *p* = 0.390) post-infection (Figure 6). However, we did observe significantly increased *nos* expression compared to controls at 24 h (*F* = 4.795, *p* = 0.014) in *A. stephensi* derived from larvae treated with 1 μM ABA (Tukey *p* = 0.041) or 100 μM ABA (Tukey *p* = 0.016) but not 10 μM ABA (Tukey *p* > 0.05; Figure 6), effects that matched patterns of infection intensity (Figure 2). At 20 h post-infection in *A. stephensi*, *P. yoelii* parasites are present as mature ookinetes, with some in the midgut lumen and a large percentage crossing the midgut epithelium [48], indicating that inducible *nos* expression is associated with inhibition of early parasite development. Further, these data suggest that the transstadial effects of ABA treatment on *P. y. yoelii* 17XNL infection in *A. stephensi* are similar in pattern to provisioning of ABA into a *P. falciparum*-infected bloodmeal, where transient repression of *ilp3* and *ilp4* was associated with early (4–6 h) inducible *nos* and NO synthesis that were cause-and-effect with repression of parasite infection [24,25].

## 4. Discussion

Understanding the mosquito–parasite relationship is critical to the pursuit of reducing malaria burden in endemic areas, but also provides insights into the conservation of innate immune mechanisms and connections between immunity and metabolism that are evident in organisms across evolutionary time. Here, we continued our analyses of the effects of the ancient stress signaling molecule ABA on *A. stephensi* physiology, specifically the response to malaria parasite infection.

In previous work, we showed that, relative to controls, ABA added to rearing water at concentrations as low as 1 μM accelerated *A. stephensi* larval development, increased pupal mortality, decreased adult female lifespan and decreased fecundity through three gonotrophic cycles in adult females derived from treated larvae [20]. With the present study, we have added to these effects increased resistance to parasite infection in adult female *A. stephensi* derived from ABA-treated larvae. Collectively, these observations have multiple touchpoints to vectorial capacity, which describes the potential for mosquitoes to transmit pathogens based on mosquito survivorship, density, fecundity, infection competence and tendency to take a second bloodmeal. The potential impact of ABA on vectorial capacity may be extended by the ingestion of ABA in a bloodmeal [24,25], which reduces parasite infection in adult female *A. stephensi* through mechanisms that correspond to the transstadial effects of ABA reported here.

To better understand the association of ABA larval treatment with reduced parasite infection and *Vg* expression in adult female *A. stephensi*, we examined the expression of *ilps*. Across multiple mosquito species, including *A. stephensi*, ILP3 and ILP4 stimulate synthesis of ovarian ecdysteroid, which is converted to 20E to induce Vg synthesis [49,50,51]. Ingestion of ABA by adult female *A. stephensi* transiently reduced *ilp3* and *ilp4*, which we previously associated not only with altered immunity, but also with changes in metabolism and stress responsiveness [25]. In particular, we noted that ABA treatment transiently increased activation of glycogen synthase kinase-3 and transiently decreased AMP kinase activity in *A. stephensi*, patterns consistent with the repression of ATP-generating catabolic reactions and mitochondrial biogenesis as well as inhibition of ATP-consuming biosynthetic processes [25]. These changes are typical of an energy-rich state and biology that could explain the global repression of *ilp* expression observed in adult female *A. stephensi* derived from ABA-treated larvae.

In *A. aegypti,* CRISPR-Cas9 depletion of ILPs led to differing levels of lipid (TAG) storage. TAG was increased with reduced *ilp5* and *ilp4* expression but decreased with reduced *ilp2* and *ilp6* expression [52]. We observed no changes in TAG levels through the first four days post-eclosion in female *A. stephensi* derived from ABA-treated larvae (Figure 3) despite significant reductions in expression of *ilps* 1–4 during this time (Figure 5). TAG is included among a group of four lipids that are limiting for malaria parasite development in the mammalian host [53] and this nutrient is similarly limiting in the mosquito host, where it is rapidly absorbed from the midgut for egg production [54], justifying our focus on TAG levels beyond the known metabolic relationship to ILPs. Given that *A. gambiae* Lp transports a broader range of neutral lipids than does *A. aegypti* Lp [55], with TAG representing <10% of Lp-associated neutral lipids in *A. gambiae* [56], it may be possible to use an anopheline model, perhaps with a push-and-pull approach with ABA, to explore more fully which Lp cargo lipids are most important for parasite development in the mosquito host.

## 5. Conclusions

The transstadial effects of ABA on *A. stephensi* likely derive from multiple impacts to metabolism and immunity to parasite infection, so the dose, duration and developmental timing of natural exposure to ABA are likely important variables to consider in defining the possible range of ABA effects on mosquito biology. The release of ABA by plants and other organisms in natural sources of water, however, is poorly studied in the context of effects on the biology of associated animal species, so these studies face some challenges. We have detected consistent, low micromolar levels of ABA in water through 48 h after inundation of plant material in the lab [20], suggesting that, despite its light sensitivity, ABA can persist under ambient temperature and lighting conditions. Further, ABA biosynthesis in plants is inducible and can vary by species [21], suggesting that ABA in both natural and container breeding sites that differ in composition of plant matter, algae and fungi could contribute to environmental effects on anopheline biology, ranging from oviposition to development to pathogen transmission. Accordingly, a better understanding of these intersections of ecology and ABA-dependent physiology in complex natural environments could lead to better biorational strategies for both mosquito and malaria control.
